# Association between respiratory diseases and molar-incisor hypomineralization: A systematic review and meta-analysis

**DOI:** 10.3389/fmed.2022.990421

**Published:** 2022-12-16

**Authors:** Yago Gecy de Sousa Né, Deborah Ribeiro Frazão, Géssica de Oliveira Lopes, Nathália Carolina Fernandes Fagundes, Renata Duarte Souza-Rodrigues, Francisco Wanderley Garcia Paula-Silva, Lucianne Cople Maia, Rafael Rodrigues Lima

**Affiliations:** ^1^Laboratory of Functional and Structural Biology, Institute of Biological Sciences, Federal University of Pará, Belém, PA, Brazil; ^2^School of Dentistry, Faculty of Medicine and Dentistry, College of Health Sciences, University of Alberta, 5528 Edmonton Clinic Health Academy, Edmonton, AB, Canada; ^3^School of Dentistry of Ribeirão Preto, University of São Paulo, São Paulo, Brazil; ^4^Department of Pediatric Dentistry and Orthodontics, School of Dentistry, Federal University of Rio de Janeiro, Rio de Janeiro, Brazil

**Keywords:** humans, molar-incisor hypomineralization, dentition permanent, tooth demineralization, respiratory tract diseases

## Abstract

The molar-incisor permineralização (MIH) is a qualitative enamel developing abnormality involving the occlusal and/or incisal third of one or more molars or permanent incisors, caused by systemic factors. Several systemic disorders and environmental factors, such as respiratory diseases, have been reported as probable causes of MIH. Thus, this work aimed to evaluate whether respiratory diseases and MIH are associated. The searches were carried out in electronic databases, including PubMed, Scopus, Web of Science, the Cochrane Library, LILACS, OpenGrey, and Google Scholar. The acronym PECO was used, in which the P (population) was humans in permanent dentition stage; (E-exposure) molar-incisor hypomineralization; (C-comparison) reference population and (O - outcome) respiratory diseases. After the search retrieval, the duplicates were removed, and the articles were evaluated by title and abstract; then, the papers were read and thoroughly assessed. After selection, the risk of bias assessment was performed using the Newcastle-Ottawa Scale (NOS) for observational studies. The Grading of Recommendations, Assessment, Development, and Evaluation (GRADE) tool was used to assess the level of evidence. Three thousand six hundred and sixty six were found through the searches. After exclusion by duplicates, title, abstract, and full-reading, 13 articles remained. The articles included in this review evaluated the association of MIH with asthma, tonsilitis, pneumonia, and bronchitis. Most reports showed a low risk of bias. When exploring through GRADE, a very low level of evidence was found. We observed that the included studies showed that children with MIH had more respiratory diseases than the group that did not have MIH.

**Systematic review registration:**

https://osf.io/un76d.

## Introduction

Several dental enamel developmental abnormalities exist, including amelogenesis imperfecta and dental fluorosis. The molar-incisor hypomineralization (MIH), associated with problems in some stages of enamel production (presecretory, secretory, transition, maturation, and post-maturation), has been described in recent years. MIH is the most prevalent among these enamel defects, yet little is known about its pathogenesis (1, 2).

MIH is a qualitative enamel developmental disorder involving the occlusal and/or incisal third of one or more molars or permanent incisors resulting from systemic factors (1, 3). Epidemiological studies indicate a significant variation in the prevalence of MIH depending on the region or group considered, ranging from 2.9 to 44% in different locations, with an overall estimate of 14.2% prevalence worldwide (4–7).

MIH can cause minor to severe changes in the enamel, which might vary even within the same person (8, 9). The intensity spectrum ranges from small white, yellow or brown demarcated opacities to significant defects involving post-eruptive breakdown (PEB) that might include large portions of the crown and cusp region (10). In the latter case, the enamel is lost after tooth eruption, exposing the underlying dentin and favoring dentinal sensitivity, as well as the development of caries lesions (11–15). The affected enamel shows damage in its mechanical properties due to changes in the conformation of the mineralized crystals and sheaths of the enamel prisms. These modifications culminate in reduced hardness and elasticity compared to normal enamel (2, 16, 17).

Although the literature presents several conditions associated with MIH, there is no consensus about its etiology (18–20). It is believed that MIH is a multifactorial disease with systemic, environmental, and hereditary factors possibly influencing the enamel maturation process (1, 21). Genetic conditions, malnutrition, use of antibiotics, chickenpox, and respiratory diseases have been reported as examples of probable causes of MIH (22–25).

In addition, dental amelogenesis, which is the process by which enamel is formed, is split into three stages: secretory, transition, and maturation. At certain moments of the maturation process, failures may occur that lead to molar-incisor hypomineralization in both deciduous and permanent dentition, since amelogenesis occurs independently in each tooth germ (22). That's why children are more susceptible to the development of hypomineralization due to systemic disturbances (22).

In this context, the prevalence of respiratory diseases in early childhood becomes alarming. A study conducted by the Global Burden of Disease (26) in 195 countries showed that respiratory tract infections are one of the leading causes of early mortality. Although there are recent studies (27, 28) describing the association of MIH with various diseases, there is still no systematic review investigating the association with only respiratory diseases, to better clarify the influence of these respiratory diseases on the onset of MIH. Thus, this systematic review sought to bring together studies that assessed the presence of respiratory diseases in individuals with and without MIH and analyze the association between these conditions.

## Materials and methods

### Registration

This systematic review was delineated following the Preferred Reporting of Systematic Review and Meta-analyses (PRISMA) (29) and registered with Open Science Framework under the URL https://osf.io/un76d.

### Eligibility criteria

This review aimed to elucidate the question: “Is there an association between molar-incisor hypomineralization and respiratory diseases?” The eligibility criteria were carried out according to the PECO strategy. It symbolizes (P-population) humans in the permanent dentition stage; (E-exposure) molar-incisor hypomineralization; (C-comparison) comparative population; and (O-outcome) respiratory disorders. Observational studies that fit the PECO were included.

Case reports, descriptive, opinion, technical, animal, and *in vitro* studies were excluded. The null hypothesis of this study is that there is no relationship between the presence of molar-incisor hypomineralization and respiratory diseases.

### Research strategy and study selection

The searches were performed in the following electronic databases: PubMed, MEDLINE, Latin American and Caribbean Health Sciences Literature database (LILACS), Scopus, Web of Science, and The Cochrane Library. OpenGrey and Google Scholar were used as gray literature. The searches were carried out until June of 2022. There was no linguistic or year restriction on demand. Medical Subject Headings (MeSH) and free terms were combined according to the syntax rule for each database. Terms related to molar-incisor hypomineralization, and diseases related to the respiratory tract in humans were searched. The search strategy adopted for each database is explicit in Supplementary Table 1.

The selection of studies took place first through evaluating the title and abstract, considering the eligibility criteria by two independent reviewers, GOL and YGSN; then, each article had its full text revised following the same protocol. The kappa test statistic for the reliability assessment was 0.99 with a *P*-value of 0.001, showing total concurrence between the two reviewers. If needed, discrepancies between reviewers were assessed by a third appraiser (RRL).

After selecting the studies, alerts were made in each database to include new studies published after the search date. A manual search of the references of the definitive studies was also carried out to include more studies that fit the criteria of this study. After searching, the citations found in each database were exported to reference management software (EndNote, X9 version, Thomson Reuters, Philadelphia, United States), and duplicated results were excluded.

### Data extraction

After selecting the studies, data related to the country, year, type of study, sample characteristics (origin and size), mean age, MIH evaluation, respiratory disease evaluation, results, and statistical analysis were extracted from all studies (Table 1). Two reviewers (GOL and YGN) performed this step and a third reviewer was checked in case of disagreement (RRL).

**Table 1:** Summary of characteristics and results of the included studies.

**Author/Country/Year/ Study design**	**Sample**			**MIH evaluation**	**Respiratory disease evaluation**	**Results**
	**Source**	***n***	**Age^a^**			
Durmus et al. (30); case-control N falou de antibiotic	Children attending the Department of Pediatric Dentistry at the Dental School of Marmara University	Total: 107 With MIH: 54 Without MIH: 53	With MIH: 9.9 ± 1.7 years Without MIH: 10.08 ± 2.25	EAPD	Questionnaire	Significant differences between groups were observed in the numbers of children who had asthma before the age of 3 years (*p* = 0.050).
Lygidakis et al. (23); case-control N falou de antibiotic	Patients of the Community Dental Center for Children in Athens	Total: 720 With MIH: 360 Without MIH: 360	With MIH: 8.17 ± 1.38 Without MIH: NI	Oral examination	Interview	Upper and lower respiratory medical problems were reported as postnatal potential etiological factor in MIH (88/162). Prevalence of respiratory problems reported: Bronchitis (5.8%), Asthma (4.1%), Bronchiolitis (1.9%), Laryngitis (1.6%), and Tonsillitis (1.38%)
Pitiphat et al. (31); cross-sectional	Students of five primary schools in urban areas of Khon Kaen District, Thailand	Total: 282 With MIH: 78 Without MIH: 204	8.0 ± 0.5	EAPD	Interview	MIH was observed more frequently in children with asthma compared with those without (52.9 vs. 26.0%). Pneumonia was found equally between the groups (28.6 vs. 27.6)
Sönmez et al. (32); cross-sectional	Students of 21 primary schools located in the urban areas of the five central municipalities of Ankara, Turkey.	Total: 3,827 With MIH: 301, Without MIH: 3,526	with MIH: 9.55 ± 2.5 years without MIH: NI	Oral examination following the suggestions of FDI Working Group (Commission on Oral Health, 1992)	Questionnaire	MIH was found to be associated with pneumonia. Asthma and respiratory tract infection were not associated with MIH.
Souza et al. (33); cross sectional	Students of public schools, in urban and rural areas of Botelhos, State of Minas Gerais, Brazil. The town	Total: 903 With MIH: 182 Without MIH: 721	9	EAPD	Questionnaire	Throat infection was linked to MIH. Pneumonia, rhinitis and bronchitis were not associated with MIH.
Beentjes et al. (34); case control N falou	Children of Amsterdam area.	Total; 45 With MIH: 24 Without MIH: 21	9.9 ± 2.02	NI	Questionnaire	Pacients with MIH: pneumonia 21%, airway infection 8%, cara 13%, asthma 8%
Ahmadi et al. (24); case control	Students of four elementary schools of Zahedans disctrict	Total: 373 With MIH: 55 Without MIH: 318	7–9	Oral examinations using DDE index	Questionnaire	Postnatal factors such as asthma were higher in MIH affected children than in normal children.
Allazzam et al. (35); cross sectional	Pacients of Pediatric Dental Clinics, Faculty of Dentistry, KAU, Jeddah, Saudi Arabia,	Total: 267 With MIH: 23 Without MIH: 244	8–12	Oral examination	Questionnaire	Children with MIH had significantly more episodes of upper respiratory tract infections including adenoiditis, tonsillitis, or asthma.
Ghanim et al. (36); case control	Students of schools in Mosul city, Iraq	Total: 823 With MIH: 153 Without MIH: 670	10–12	EAPD	Questionnaire	The risk of MIH was significantly more likely to happen following acute health illnesses: pneumonia (OR, 2.28), tonsillitis (OR, 4.00) and pneumonia (OR, 9.37)
de Lima et al. (37); cross-sectional	Students of public and private schools of Teresina city, Piauí, Brazil	Total: 594 With MIH: 109 Without MIH: 485	11–14	EAPD	Questionnaire	Respiratory distress in postnatal period was not associated with MIH
Muratbegovic et al. (38); case-control	Students of Schools of nine cities in Boznia and Herzegovina	Total: 530 With MIH: 69 Without MIH: 491	12	EAPD	Questionnaire	Correlation between tonsillitis and MIH (*p* = 0.06) suggested that patients who had frequent tonsillitis were more likely to present MIH.
Whatling and Fearne et al. (39); case control	Department of Pediatric Dentistry at The Royal London Hospital	Total: 109 With MIH: 57 Without MIH: 52	With: 8.51 Without: 8.85	NI	Interview	No correlation found between asthma and MIH (*p* = 0.856).
Wuollet et al. (40)/ Finland	Schools from two rural Finnish towns, Lammi and Jalasj€arvi	Total: 287 With MIH:33 Without MIH: 254	10.4	EAPD	Medical records	Respiratory infectious illnesses were not significantly associated with MIH.

EAPD, Oral examination with European Academy of Paediatric Dentistry criteria; NI, not informed. ^*a*^Age was reported as mean§SD, median or minimal age reported in the inclusion criteria.

### Risk of bias

The New Castle-Ottawa scale for observational studies was used to assess the quality of the articles included (41). In this scale, the methodological quality was evaluated by a star system in three domains: selection of participants, comparability of study groups, and determination of the results of interest. In the first section, the study is evaluated regarding the case definition, the representativeness of the cases, and the selection and definition of the controls. The second domain evaluates the comparability of cases and controls based on the design or analysis. The exposure section analyzes the ascertainment of exposure, the non-response rate, and whether the study used the same method for cases and controls. The studies were evaluated, reaching a total score of 9 stars at most, four stars for selection, two for comparability, and three for the outcome (Tables 2, 3).

**Table 2:** Newcastle-Ottawa for case-control studies.

**Case control**	**Durmus et al. (30)**	**Lygidakis et al. (23)**	**Beentjes et al. (34)**	**Ahmadi et al. (24)**	**Ghanim et al. (36)**	**Muratbegovic et al. (38)**	**Whatling and Fearne (39)**
**Selection**							
1) Is the case definition adequate?	*	*	–	*	*	*	*
2) Representativeness of the cases	*	*	–	*	*	*	*
3) Selection of controls	*	*	–	–	*	*	*
4) Definition of controls	*	*	–	*	*	*	*
**Comparability**							
1) Comparability of cases and controls on the casis of the design or analysis	**	**	*	*	*	**	**
**Exposure**							
1) Ascertainment of exposure	*	*	–	*	*	*	-
2) Same method of ascertainment for cases and controls	*	*	–	*	*	*	*
3) Non-response rate	–	*	–	–	*	*	*

The * and ** symbols indicate score that each article received in the quality assessment.

**Table 3:** Newcastle-Ottawa for cross-sectional studies.

**Cross sectional**	**Pitiphat et al. (31)**	**Sönmez et al. (32)**	**Souza et al. (33)**	**Allazzam et al. (35)**	**de Lima et al. (37)**	**Wollet et al. (40)**
**Selection (5 stars max)**						
1) Is the case definition adequate?	*	*	*	*	*	*
2) Sample	-	*	-	-	*	*
3) Non-respondents	*	*	*	-	*	*
4) Ascertainment of the exposure (risk factor)	**	**	**	**	**	**
**Comparability (2 stars max)**						
1) The subjects in different outcome groups are comparable, based on the study design or analysis. confounding factors are controlled	*	**	*	*	**	*
**Outcome (3 stars max)**						
1) Ascertainment of outcome	**	**	-	-	-	**
2) Statistical test	*	*	*	*	*	*

The * and ** symbols indicate score that each article received in the quality assessment.

### Quantitative analysis

Only the cross-sectional studies were included in the quantitative analysis to evaluate the prevalence of respiratory diseases in an MIH population. Three studies have been excluded due to methodological heterogeneity (the methodology of the articles being very different from the others included in the meta-analysis).

Data from the included studies were analyzed using Review Manager software (Review Manager v. 5.3, The Cochrane Collaboration; Copenhagen, Denmark). Five independent meta-analyses were performed to evaluate the prevalence of asthma (1), pneumonia (2), tonsilitis (3), bronchitis (4), and rhinitis (5) among control and MIH patients. The total events were entered in each analysis, and a fixed-effects model was adopted. The Odds ratio with a 95% confidence interval (CI) was used to report the outcomes. If any of the selected papers missed part of the information required for the meta-analysis, the authors were contacted to provide the missing information (42).

Heterogeneity was tested using the I2 index, and, if possible, sensitivity analyses were conducted to estimate and verify the influence of studies, one by one, when the heterogeneity was substantial or considerable (50 to 100%) (www.training.cochrane.org/handbook).

### Certainty of evidence

The overall certainty of evidence was presented using the Grading of Recommendation, Assessment, Development, and Evaluation (GRADE) tool (43). Evidence from observational studies was initially classified as low quality but can be increased according to the methodological design, risk of bias, consistency, and directness. Five subgroups were created following the results from meta-analyses: prevalence of asthma (1), the prevalence of pneumonia (2), the prevalence of tonsilitis (3), the prevalence of bronchitis (4), and prevalence of rhinitis (5).

## Results

### Selection and characteristics of the studies

**Figure 1:**
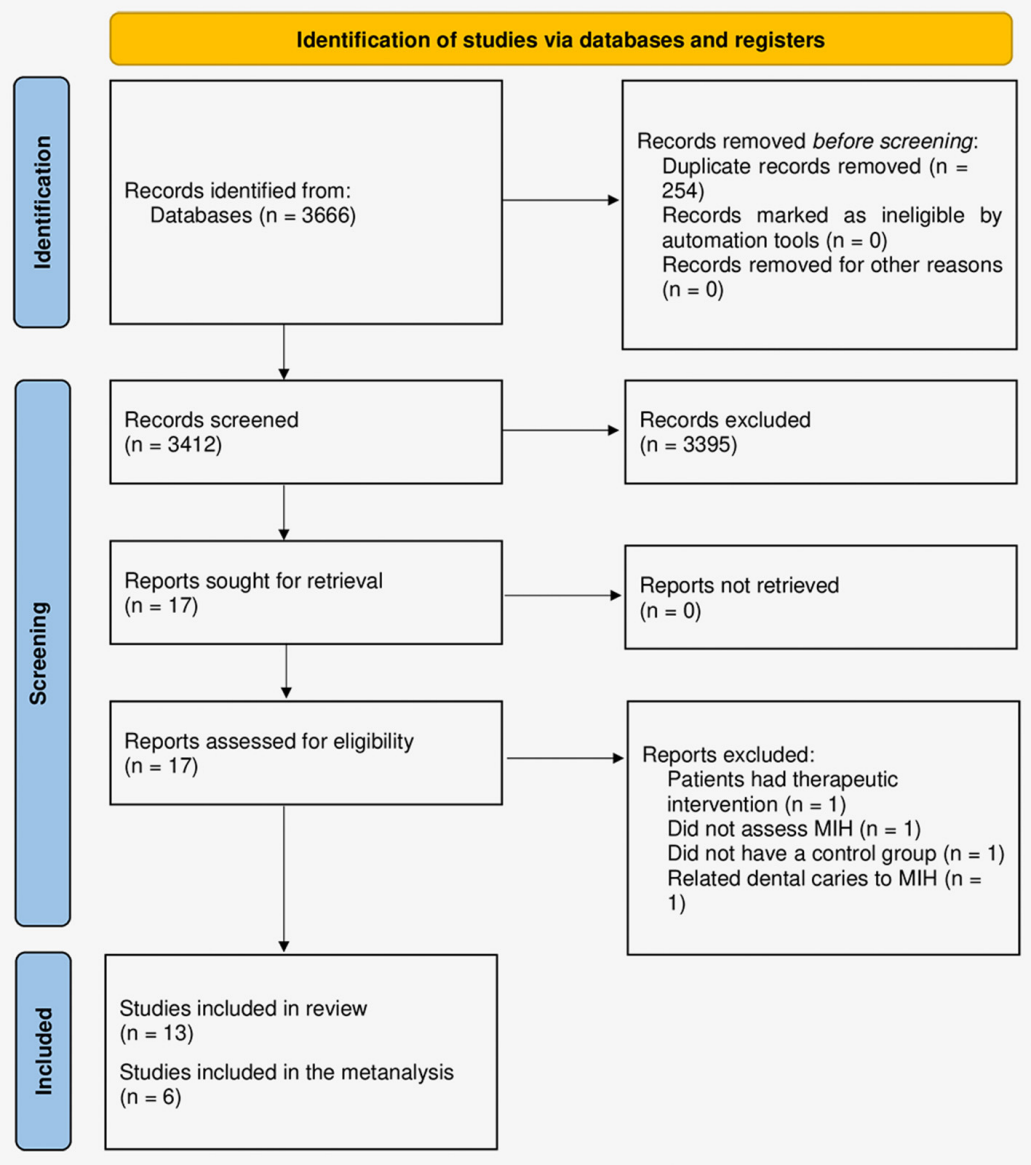
Flowchart of the study selection process according to the PRISMA protocol (29).

Three thousand six hundred sixty-six articles were found after the searches. After excluding duplicates, 3,412 remained and had their titles and abstracts evaluated, in which 17 articles had their texts read in full. In this phase, four studies were excluded 1 study was excluded for having a therapeutic intervention (44), 1 for not assessing MIH (45), 1 had no control group (46), and 1 for relating dental caries to MIH (47), resulting in 13 final articles (Figure 1).

Therefore, thirteen articles were included in this systematic review (23, 24, 30–40). Seven of them were case-control (23, 24, 30, 34, 36, 38, 39) and six were cross-sectional studies (31–33, 35, 37, 40) (Table 1). A total of 8,897 individuals were evaluated, of which 1,498 were included in the group with MIH and 7,399 in the group without MIH, with a mean age of the participants of 9.78 years. Three studies were performed in dental departments of universities, six in schools, and two did not specify the location of recruitment and analysis of individuals.

Regarding the MIH diagnosis, seven studies used the criteria of the European Academy of Pediatric Dentistry (EAPD) (30, 31, 33, 36–38, 40), one study used the modified version of the Developmental Defects of Enamel (DDE) Index (24), one study classified injuries according to the criteria of the FDI Working Group, Comission on Oral Health, 1992; (32). Two studies used previously trained examiners to perform the MIH screening, and two did not specify the method for diagnosing MIH injuries (23, 34, 35, 39). To assess the history of respiratory diseases in the study participants, nine studies evaluated questionnaires answered by those responsible for their medical history (24, 30, 32–38), three studies conducted an interview with those responsible (23, 31, 39) and one study carried out an analysis of medical records (40). Of the respiratory diseases observed in the studies, asthma, pneumonia, tonsillitis and bronchitis were found more frequently in the individuals participating.

Among the thirteen articles included in this review, ten (23, 24, 30–32, 34, 35, 37–39) observed asthma as a respiratory disease present in the individuals studied. Four had a significantly higher prevalence in individuals with MIH than patients without the condition. In descriptive studies, Lygidakis observed a prevalence of 4.1% of the disease, which ranged from 0 to 4.1% (23).

Seven studies reported the presence of pneumonia (31–33, 36–38). Among those, only two found an association with MIH (32, 34). Beentjes hypothesizes that lack of oxygen is involved in the development of MIH.

Tonsillitis has been reported in eight studies (23, 30–33, 35, 36, 38). Only three of these showed significant results concerning the group without MIH, and one of these showed a statistically significant borderline result (*p* = 0.06) (38). Sönmez et al. (32) described that 7–12 years old children with tonsillitis had a 1,136 Odds Ratio (OR; 95% CI: 0.865–1.493, 0.359) of having MIH. On the other hand, Souza et al. (33) observed that 6–12 years old children with tonsillitis from urban and rural areas in a Brazilian city had an OR of 0.80 (95% CI: 0.51–1.27, 0.359) of having MIH.

Bronchitis was observed in 5 studies, with only one showing significance between the group of children with MIH and without MIH, with an OR of 1,284 (95% CI: 0.965–1.708, 0.086).

### Qualitative assessment of studies and risk of bias

Among the studies included, thirteen showed good quality according to the assessed domains, earning 6 to 10 stars (23, 24, 30–34, 36–40). Problems were observed in the definition of the cases of MIH, representativeness, selection and definition of controls for the case-control studies. In cross-sectional studies, it was found that failures in the sampling method, correspondence between groups and verification of the outcome impair the general quality of the articles. The results of this quality assessment are shown in Tables 2, 3.

### Quantitative analysis

To evaluate the prevalence of respiratory diseases in an MIH population, only the cross-sectional studies were included in the quantitative analysis (24, 31–33, 36, 37). Two case-controls (34, 39) and one RCT (30) study have been excluded due to methodological heterogeneity.

#### Prevalence of asthma

**Figure 2:**
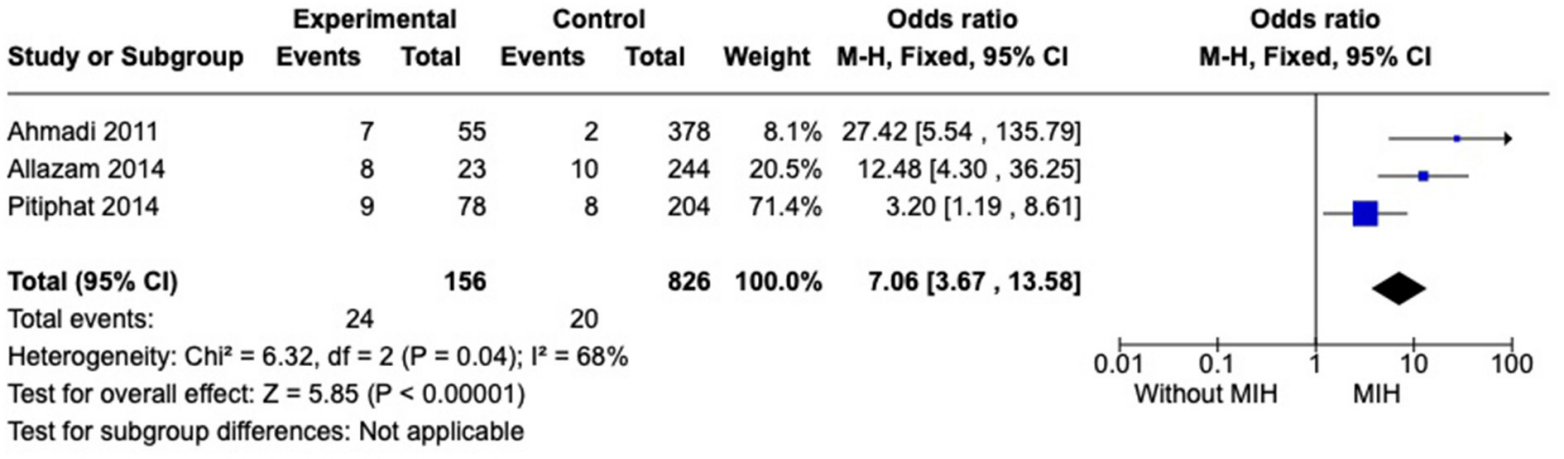
Forest plot of meta-analysis for the prevalence of asthma among subjects with MIH. CI, confidence interval.

Five studies were included in this analysis. However, a high statistical heterogeneity was observed among studies. To reduce heterogeneity, a sensitivity analysis was performed. Removing studies, one by one, the heterogeneity ranges from 86 to 68%. Therefore, two studies, Sönmez et al. (32) and de Lima et al. (37) were excluded, and the *I*^2^ = 68% was considered. As a result, we observed that individuals with MIH (*n* = 156) showed a higher prevalence of asthma than subjects without MIH (*n* = 826), OR = 7.06 [3.67, 13.58] (Figure 2).

#### Prevalence of pneumonia

**Figure 3:**
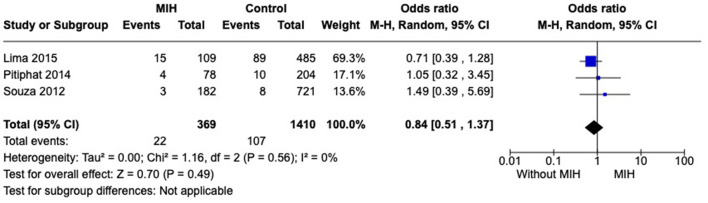
Forest plot of meta-analysis for the prevalence of pneumonia among subjects with MIH. CI, confidence interval.

Five studies were included in this analysis. However, a high statistical heterogeneity was observed among studies. To reduce heterogeneity, a sensitivity analysis was performed. Removing studies, one by one, the heterogeneity ranges from 82 to 0%. Therefore, two studies, Sönmez et al. (32) and Ghanim et al. (36) were excluded, and the *I*^2^ = 0% was considered. No difference was observed regarding prevalence of pneumonia when comparing groups with and without MIH across studies (*p* = 0.49) (Figure 3).

#### Prevalence of tonsillitis

**Figure 4:**
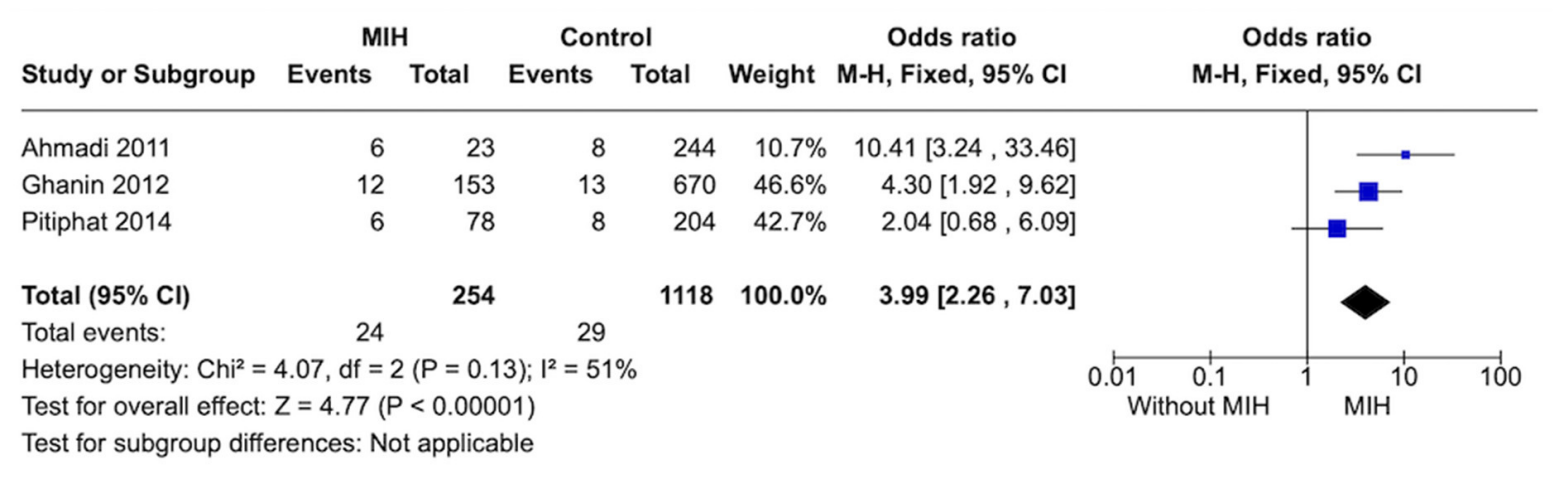
Forest plot of meta-analysis for the prevalence of tonsilitis among subjects with MIH. CI, confidence interval.

Four studies evaluated the prevalence of tonsillitis. After performing the analysis, a high statistical heterogeneity was observed among studies. To reduce heterogeneity, a sensitivity analysis was performed. Removing studies, one by one, the heterogeneity ranges from 83 to 51%. Therefore, Sömnez et al. (32) was excluded, and the I2 = 51% was considered. As a result, individuals with MIH (*n* = 254) showed a higher prevalence of tonsillitis than subjects without MIH (*n* = 1,118), OR = 3.99 [2.26, 7.03] (Figure 4).

#### Prevalence of bronchitis

**Figure 5:**
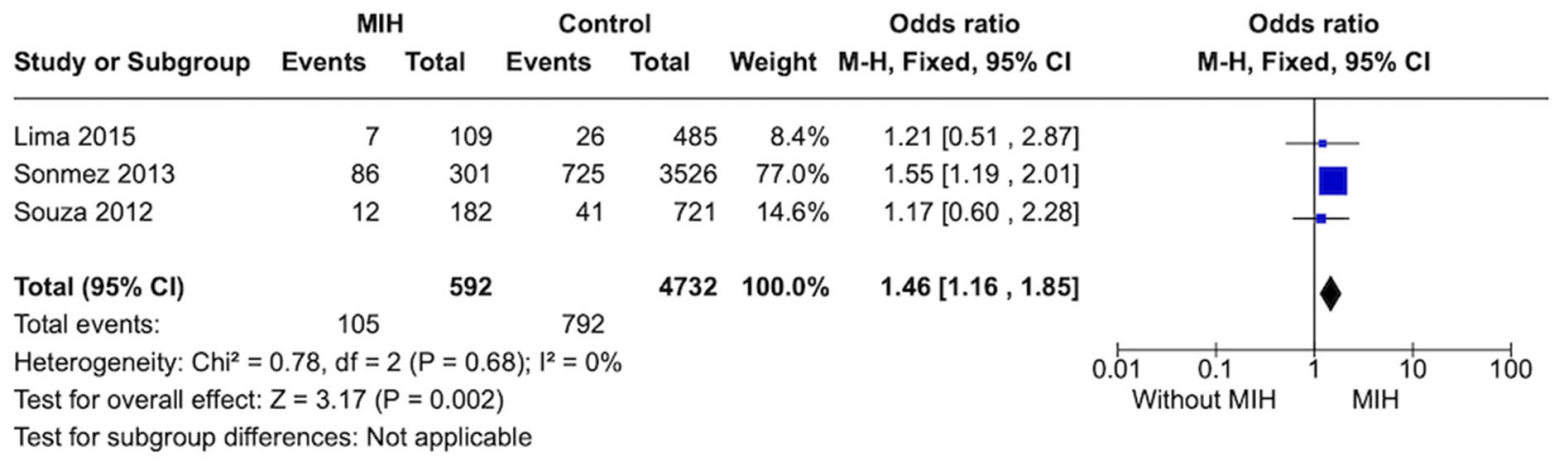
Forest plot of meta-analysis for the prevalence of bronchitis among subjects with MIH. CI, confidence interval.

Three studies were included in this analysis. Individuals without MIH (*n* = 4,732) showed a lower prevalence of bronchitis than subjects with MIH (*n* = 592), OR = 1.46 [1.16, 1.85]. A low heterogeneity was observed between studies (Figure 5).

#### Prevalence of rhinitis

**Figure 6:**
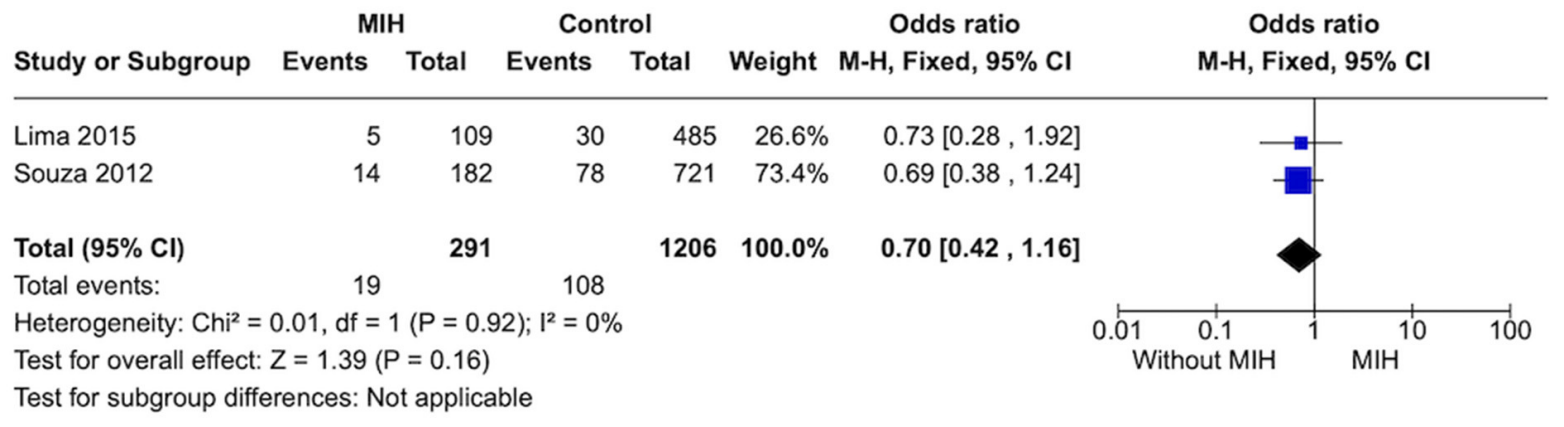
Forest plot of meta-analysis for the prevalence of rhinitis among subjects with MIH. CI, confidence interval.

Two studies were included in this analysis. Individuals without MIH (*n* = 1,206) showed no strong evidence difference on the prevalence of rhinitis when compared to subjects with MIH (*n* = 592) (*p* = 0.16). A low heterogeneity was observed between studies (Figure 6).

### Certainty of evidence

In the GRADE analysis, a very low certainty of the evidence was observed among all outcomes evaluated (Table 4). For instance, the overall certainty of evidence from the Asthma metanalysis was considered “very low” because Allazzam et al. (35) and de Lima et al. (37) showed a poor methodological quality and a moderate statistical heterogeneity (I^2^ = 68%) was detected. Moreover, the risk of bians in the Pneumonia investigation was serious because one study (37) showed a poor methodological quality. Tonsilitis' level of evidence had serious problems in risk of bias, because Allazzam et al. (35) showed a poor methodological quality, and in inconsistency, because a moderate statistical heterogeneity (I^2^ = 51%) was detected. Finally, both the Bronchitis and Rhinitis metanalyses indicated a very low level of evidence because they raised some serious concerns about bias due to de Lima et al. (37) study.

**Table 4:** Certainty of the evidence evaluation (GRADE approach).

**MIH compared to control for respiratory diseases**											
**Bibliography:**											
**Certainty assessment**							**Summary of findings**				
**Participants (studies) Follow-up**	**Risk of bias**	**Inconsistency**	**Indirectness**	**Imprecision**	**Publication bias**	**Overall certainty of evidence**	**Study event rates (%)**		**Relative effect (95% CI)**	**Anticipated absolute effects**	
							**With control**	**With MIH**		**Risk with control**	**Risk difference with MIH**
**Asthma**											
5,403 (5 observational studies)	Very serious^a^	serious^b^	Not serious	Not serious	None	⊕○○○ Very low	136/4,837 (2.8%)	43/566 (7.6%)	OR 2.22 (1.54 to 3.20)	28 per 1,000	32 more per 1,000 (from 15 more to 57 more)
**Pneumonia**											
6,429 (5 observational studies)	Serious^c^	Not serious	Not serious	Not serious	None	⊕○○○ Very low	318/5,606 (5.7%)	71/823 (8.6%)	OR 1.69 (1.28 to 2.23)	57 per 1,000	36 more per 1,000 (from 15 more to 62 more)
**Tonsilitis**											
1,372 (3 observational studies)	serious^d^	Serious^e^	Not serious	Not serious	None	⊕○○○ Very low	29/1,118 (2.6%)	24/254 (9.4%)	OR 3.99 (2.26 to 7.03)	26 per 1,000	70 more per 1,000 (from 31 more to 132 more)
**Bronchitis**											
5,324 (3 observational studies)	Serious^c^	Not serious	Not serious	not serious	none	⊕○○○ Very low	788/4,732 (16.7%)	105/592 (17.7%)	OR 1.46 (1.16 to 1.85)	167 per 1,000	59 more per 1,000 (from 22 more to 103 more)
**Rinitis**											
1,497 (2 observational studies)	Serious^c^	Not serious	Not serious	Not serious	None	⊕○○○ Very low	108/1,206 (9.0%)	19/291 (6.5%)	OR 0.70 (0.42 to 1.16)	90 per 1,000	25 fewer per 1,000 (from 50 fewer to 13 more)

CI, confidence interval; OR, odds ratio. ^a^de Lima et al. [*32*] and Allazzam et al. (35) showed a poor methodological quality. ^b^A moderate statistical heterogeneity (I^2^ = 68%) was detected. ^c^de Lima et al. (37) showed a poor methodological quality. ^d^Allazzam et al. (35) showed a poor methodological quality. ^e^A moderate statistical heterogeneity (I^2^ = 51%) was detected.

## Discussion

Among the selected articles, the primary respiratory diseases that appeared as postnatal events related to MIH were asthma, pneumonia, tonsillitis, and bronchitis. Nine out of thirteen articles included in this systematic review reported an association between MIH and respiratory diseases. Eight papers were included in the statistical analysis through meta-analysis, evaluating some respiratory diseases. Despite the study's limitations and the low certainty of evidence demonstrated by GRADE, an overall examination of the studies reveals an association between MIH and respiratory disorders, as shown by the meta-analysis results.

MIH is a defect of multifactorial origin that occurs in dental enamel. The first permanent molar enamel formation (amelogenesis) occurs from the twenty-eighth gestational week. The ameloblast, cell responsible to produce enamel, is among the most sensitive cells in the human body: if its function is temporarily or permanently interrupted depending on the time of injury, hypoplasia or hypomineralization of the enamel might occur (48–50). Some diseases in the prenatal period and infancy can lead to problems in the supply of oxygen to ameloblasts, which causes a loss of the mineral-secreting capacity of these cells (39, 51, 52). Premature births, problems in pregnancy, use of antibiotics, some respiratory diseases (asthma; bronchitis), and some diseases such as chickenpox, otitis, fever, and contact of both the hand and the child with environmental toxins, are highly related to the alteration of the mineral synthesis of ameloblasts causing defects in tooth enamel, such as MIH (39, 51).

One of the main hypotheses for the emergence of MIH comes from the lack of oxygen, mainly through diseases that occurred in the perinatal period, such as asthma, bronchitis, pneumonia, rhinitis, which may cause an imbalance of oxygen in the head and neck region (34, 53, 54). Some conditions, such as an respiratory acidosis due to abnormal oxygen levels due to hypoventilation that occurs in respiratory diseases, may change the pH values of the enamel matrix and results in an inhibition of the action of proteolytic enzymes. This may negatively impact in the growth of hydroxyapatite crystals (55, 56). In this systematic review, we were able to point out a direct association between MIH and respiratory diseases, as reported by Beentjes et al. (34), who showed a difference between the groups with and without MIH regarding respiratory diseases in the perinatal period in which the group with MIH had a total of 8% more cases of asthma than the group without MIH, which had no cases.

Another mechanism in MIH proposed involves extracellular disturbances that causes mineralization poisoning (57, 58). The mineralization-poisoning model involves entrapment of albumin into enamel matrix, which binds to immature enamel crystals and block the entry of mineral ions to the growth surface, resulting in chalky opacities (59).

Analyzing the process and chronology of tooth eruption is of paramount importance for the observation of some biological occurrences that environmental and genetic factors can influence, usually, at 6 months of age, the eruption of deciduous teeth begins and, on average, at 6 years of age, the eruption of teeth permanent teeth, any genetic alteration or any systemic involvement in the prenatal period or early childhood might affect the formation of mature enamel (60). In the articles retrieved in this systematic review, it is possible to observe that children who suffered some type of respiratory diseases in early childhood ended up having a greater number of cases of MIH (32, 34), the age group found in these articles was usually children aged 8 to 14 years, during which period eruption of permanent teeth is occurring (32, 34).

The meta-analyses conducted in our study showed that patients with MIH were more closely related to respiratory diseases, such as asthma, and tonsillitis. According to the GDB (26), there is a high prevalence of respiratory diseases in early childhood, which are responsible for a significant cause of early death. Respiratory diseases end up having a very big impact on children due to the little development of the immune system, the proximity between the bronchi and the trachea, and the little developed lungs (61). These characteristics in children cause a fast transmission of infectious agents in different anatomical structures and a high resistance to the total volume of inspired air, favoring the appearance of obstructive problems and deficits in physical and cognitive development (62).

Respiratory diseases are classified into high and low, both of which can be caused by viruses and bacteria. Upper airway infections can also be called upper airway infections, in which the upper airway is compromised, including rhinitis and pharyngitis (63, 64). In lower respiratory diseases, which affect the lower airways, we have pneumonia and bronchitis, which are more severe diseases that require more complex levels of care (63, 64). In this systematic review, the meta-analysis performed on rhinitis, pneumonia and bronchitis showed that there was no statistical difference between the groups, not showing an strong evidence evidence of a relationship between this diseases e and OHM.

Although we found that children with MIH had more respiratory diseases, some limitations of the studies used in this systematic review should be pointed out. When analyzing the risk of bias, ten articles showed good methodological quality, but when analyzing the level of certainty in evidence, there is very low evidence, mainly due to the high statistical heterogeneity found. The studies generally had good results with a low risk of bias, but three had problems mainly in the selection and exposure domains (34, 35, 37). Thus, this showed an altered bias in at least one of the evaluated domains and revealed a methodological flaw throughout the three articles. These methodological flaws can hinder the real vision of the results obtained throughout these articles.

The level of evidence from the joint studies carried out by GRADE was considered very low for asthma, pneumonia, and tonsillitis and moderate for bronchitis and rhinitis. This tool assesses whether the evidence from study selection is strong enough to conclude the association between MIH and respiratory disease. The main problems found were that some studies (35, 37) showed a low methodological quality, which hinders an overall analysis of the results. Also, it was possible to observe a high heterogeneity between studies, precluding the assumption that respiratory diseases in early childhood cause MIH.

It is worth noting that the systematic review is a secondary study which constructs parallel papers, evaluating their methodology, and providing them together in an extremely applicable mathematics analysis when possible. When based on randomized controlled trials, this type of study was once considered the best scientific evidence available to understand higher cognitive processes in questions about therapeutics. However, recent updates to the pyramid of scientific evidence were made. Due to certain limitations of this type of study, such as clinical, statistical, or methodological heterogeneity, both systematic review and meta-analysis were removed from the top of the pyramid and adopted as a tool to analyze other study types, assisting in the application of the reported evidence (65–68). Due to the nature of the investigation, in this systematic review and meta-analysis, we evaluated observational studies that only allow us to infer the association between MIH and respiratory diseases, not allowing us to assume causality between the events.

Furthermore, it is important to consider that although this systematic review investigates the association between respiratory diseases and MIH, it is important to note that in many of these diseases, patients may be on antibiotic therapy. Another systematic review addressing the etiology of MIH, for example, found that 5 studies included in our selected papers reported information on antibiotic use. They mentioned that although Allazzam et al. (35) found an association between antibiotic use at any time in early childhood and MIH, Pitiphat et al. (31) discovered that this association was no longer significant after controlling for confounding factors. Whatling and Fearne (39) discovered a strong correlation with amoxicillin use. Moreover, when amoxicillin was combined with other antibiotics, Souza et al. (33) found a significant association, but only in rural areas, without examining the relationship with amoxicillin itself. Finally, Ghanim et al. (36) reported that the type of antibiotic had no association, but no additional information was provided. Nevertheless, the other eight studies included in our review stated that the study collected data on specific illnesses rather than the antibiotics used to treat them, or even made no mention of antibiotic therapy.

Therefore, for further elucidation of the results obtained in this study, more retrospective studies are needed to show the severity of the infection better, the use of medications during early childhood, and especially the stage of tooth formation that occurs when the child presents respiratory disease, this being the major limitation of this study.

## Conclusion

In this systematic review, we observed that the included studies showed that children with MIH had more respiratory diseases than the group that did not have MIH. In the meta-analysis, only rhinitis had no statistical differences between the groups, and the other diseases analyzed showed higher frequency in the group with MIH. However, the studies did not show at what stage of dental formation the teeth were when these children contracted these diseases. They also presented a high heterogeneity among the included studies, and some showed flaws in their methodologies. Thus, retrospective studies that clarify the stage of tooth formation, use of medications, and severity of respiratory diseases are necessary to understand MIH's association with these respiratory diseases effectively.

## Data availability statement

The original contributions presented in the study are included in the article/Supplementary material, further inquiries can be directed to the corresponding author/s.

## Author contributions

YN, DF, GL, and RL designed the study and performed the searches, data extraction, quality assessment, analysis of results, and manuscript elaboration. YN, NF, and RL performed analysis of results and manuscript elaboration. NF performed quantitative analysis. RS-R, FP-S, LM, and RL performed analysis of results and manuscript evaluation. All authors contributed to the article and approved the submitted version.

## References

[1] Silva MJ Scurrah KJ Craig JM Manton DJ Kilpatrick N. Etiology of molar incisor hypomineralization - a systematic review. Community Dent Oral Epidemiol. (2016) 44:342–53. 10.1111/cdoe.12229 27121068

[2] Raposo F de Carvalho Rodrigues AC Lia ÉN Leal SC. Prevalence of hypersensitivity in teeth affected by molar-incisor hypomineralization (MIH). Caries Res. (2019) 53:424–30. 10.1159/000495848 30677762

[3] Weerheijm KL Jälevik B Alaluusua S. Molar-incisor hypomineralisation. Caries Res. (2001) 35:390–1. 10.1159/000047479 11641576

[4] Denis M Atlan A Vennat E Tirlet G Attal JP. White defects on enamel: diagnosis and anatomopathology: two essential factors for proper treatment. part 1 *.Int Orthod*. (2013) 11:139–65. 10.1016/j.ortho.2013.02.014 23597715

[5] Elfrink ME Ghanim A Manton DJ Weerheijm KL. Standardised studies on molar incisor hypomineralisation (MIH) and hypomineralised second primary molars (HSPM): a need. Eur Arch Paediatr Dent. (2015) 16:247–55. 10.1007/s40368-015-0179-7 25894247

[6] Zhao D Dong B Yu D Ren Q Sun Y. The prevalence of molar incisor hypomineralization: evidence from 70 studies. Int J Paediatr Dent. (2017) 28:170–9. 10.1111/ipd.12323 28732120

[7] Negre-Barber A Montiel-Company JM Catalá-Pizarro M Almerich-Silla JM. Degree of severity of molar incisor hypomineralization and its relation to dental caries. Sci Rep. (2018) 8:1248. 10.1038/s41598-018-19821-0 29352193 PMC5775201

[8] dos Santos MPA Maia LC. Molar incisor hypomineralization: morphological, aetiological, epidemiological and clinical considerations. In:Li M-Y, editor. Contemporary Approach to Dental Caries. InTech Open (2012). 10.5772/37372

[9] Baroni C Marchionni S. MIH supplementation strategies: prospective clinical and laboratory trial. J Dent Res. (2011) 90:371–6. 10.1177/0022034510388036 21149856

[10] Brook AH. Multilevel complex interactions between genetic, epigenetic and environmental factors in the aetiology of anomalies of dental development. Arch Oral Biol. (2009) 54:S3–17. 10.1016/j.archoralbio.2009.09.005 19913215 PMC2981858

[11] Kilpatrick N. New developments in understanding development defects of enamel: optimizing clinical outcomes. J Orthod. (2009) 36:277–82. 10.1179/14653120723310 19934246

[12] Elhennawy K Schwendicke F. Managing molar-incisor hypomineralization: a systematic review. J Dent. (2016) 55:16–24. 10.1016/j.jdent.2016.09.012 27693779

[13] Americano GC Jacobsen PE Soviero VM Haubek D. A systematic review on the association between molar incisor hypomineralization and dental caries. Int J Paediatr Dent. (2017) 27:11–21. 10.1111/ipd.12233 27098755

[14] Bonzanini LIL Arduim ADS Lenzi TL Hugo FN Hilgert JB Casagrande L . Molar-incisor hypomineralization and dental caries: a hierarchical approach in a populational-based study. Braz Dent J. (2021) 32:74–82. 10.1590/0103-6440202104511 35019021

[15] Duarte MBS Carvalho VR Hilgert LA Ribeiro APD Leal SC Takeshita EM. Is there an association between dental caries, fluorosis, and molar-incisor hypomineralization? J Appl Oral Sci. (2021) 29:e20200890. 10.1590/1678-7757-2020-0890 34287466 PMC8289254

[16] Jalevik B Dietz W Noren JG. Scanning electron micrograph analysis of hypomineralized enamel in permanent first molars. Int J Paediatr Dent. (2005) 15:233–40. 10.1111/j.1365-263X.2005.00644.x 16011781

[17] Schwendicke F Elhennawy K Reda S Bekes K Manton DJ Krois J. Global burden of molar incisor hypomineralization. J Dent. (2018) 68:10–8. 10.1016/j.jdent.2017.12.002 29221956

[18] Crombie F Manton D Kilpatrick N. Aetiology of molar-incisor hypomineralization: a critical review. Int J Paediatr Dent. (2009) 19:73–83. 10.1111/j.1365-263X.2008.00966.x 19250392

[19] Serna C Vicente A Finke C Ortiz AJ. Drugs related to the etiology of molar incisor hypomineralization. a systematic review. J Am Dent Assoc. (2016) 147:120–30. 10.1016/j.adaj.2015.08.011 26552335

[20] Fatturi AL Wambier LM Chibinski AC Assunção LRDS Brancher JA Reis A . systematic review and meta-analysis of systemic exposure associated with molar incisor hypomineralization. Community Dent Oral Epidemiol. (2019) 47:407–15. 10.1111/cdoe.12467 31111554

[21] Teixeira RJPB Andrade NS Queiroz LCC Mendes FM Moura MS Moura LFAD . Exploring the association between genetic and environmental factors and molar incisor hypomineralization: evidence from a twin study. Int J Paediatr Dent. (2018) 28:198–206. 10.1111/ipd.12327 28833715

[22] Alaluusua S. Aetiology of molar-incisor hypomineralisation: a systematic review. Eur Arch Paediatr Dent. (2010) 11:53–5. 10.1007/BF03262713 20403298

[23] Lygidakis NA Dimou G Marinou D. Molar-incisor hypomineralisation (MIH): a retrospective clinical study in Greek children. II possible medical aetiological factors. Eur Arch Paediatr Dent. (2008) 9:207–17. 10.1007/BF03262637 19054474

[24] Ahmadi R Ramazani N Nourinasab R. Molar incisor hypomineralization: a study of prevalence and etiology in a group of Iranian children. Iran J Pediatr. (2011) 22:245–51. Available online at: https://www.ncbi.nlm.nih.gov/pmc/articles/PMC3446062/pdf/IJPD-22-245.pdf 23056894 PMC3446062

[25] Garot E Rouas P Somani C Taylor GD Wong F Lygidakis NA. An update of the aetiological factors involved in molar incisor hypomineralisation (MIH): a systematic review and meta-analysis. Eur Arch Paediatr Dent. (2021) 23:23–38. 10.1007/s40368-021-00646-x 34164793

[26] GBD. Lower Respiratory Infections Collaborators. Estimates of the global, regional, and national morbidity, mortality, and aetiologies of lower respiratory infections in 195 countries, 1990-2016: a systematic analysis for the Global Burden of Disease Study. (2018) p. 1191–210. 10.1016/S1473-3099(18)30310-4 PMC6202443 30243584

[27] Bandeira Lopes L Machado V Botelho J Haubek D. Molar-incisor hypomineralization: an umbrella review. Acta Odontol Scand. (2021) 79:359–69. 10.1080/00016357.2020.1863461 33524270

[28] Lopes LB Machado V Mascarenhas P Mendes JJ Botelho J. The prevalence of molar-incisor hypomineralization: a systematic review and meta-analysis. Sci Rep. (2021) 11:22405. 10.1038/s41598-021-01541-7 34789780 PMC8599453

[29] Page MJ McKenzie JE Bossuyt PM Boutron I Hoffmann TC Mulrow CD . The PRISMA 2020 statement: an updated guideline for reporting systematic reviews. BMJ. (2021) 372:n71. 10.1136/bmj.n71 33782057 PMC8005924

[30] Durmus B Abbasoglu Z Peker S Kargul B. “Possible medical aetiological factors and characteristics of molar incisor hypomineralisation in a group of Turkish children/Moguci medicinski etioloski cimbenici i znacajke molarno incizivne hipomineralizacije u skupini turske djece.” *Acta Stomatologica Croatica*. (2013) 4:297–306. 10.15644/asc47/4/1 27688372 PMC4872817

[31] Pitiphat W Luangchaichaweng S Pungchanchaikul P Angwaravong O Chansamak N. Factors associated with molar incisor hypomineralization in Thai children. Eur J Oral Sci. (2014) 122:265–70. 10.1111/eos.12136 24924351

[32] Sönmez H Yildirim G Bezgin T. Putative factors associated with molar incisor hypomineralisation: an epidemiological study. Eur Arch Paediatr Dent. (2013) 14:375–80. 10.1007/s40368-013-0012-0 23860619

[33] Souza JF Costa-Silva CM Jeremias F Santos-Pinto L Zuanon AC Cordeiro RC. Molar incisor hypomineralisation: possible aetiological factors in children from urban and rural areas. Eur Arch Paediatr Dent. (2012) 13:164–70. 10.1007/BF03262865 22883354

[34] Beentjes VE Weerheijm KL Groen HJ. Factors involved in the aetiology of molar-incisor hypomineralisation (MIH). Eur J Paediatr Dent. (2002) 3:9–13. Available online at: https://www.ejpd.eu/abstract-pubmed/factors-involved-in-the-aetiologyof-molar-incisor-hypomineralisation-mih/12871011

[35] Allazzam SM Alaki SM El Meligy OA. Molar incisor hypomineralization, prevalence, and etiology. Int J Dent. (2014) 2014:234508. 10.1155/2014/234508 24949012 PMC4034724

[36] Ghanim AM Morgan MV Mariño RJ Bailey DL Manton DJ. Risk factors of hypomineralised second primary molars in a group of Iraqi schoolchildren. Eur Arch Paediatr Dent. (2012) 13:111–8. 10.1007/BF03262856 22652207

[37] de Lima MdD Andrade MJ Dantas-Neta NB Andrade NS Teixeira RJ de Moura MS . Epidemiologic Study of Molar-incisor Hypomineralization in Schoolchildren in North-eastern Brazil. Pediatr Dent. (2015) 37:513–9. Available online at: https://www.ingentaconnect.com/content/aapd/pd/2015/00000037/00000007/art00004;jsessionid=312ut1kpdnucv.x-ic-live-01 26883608

[38] Muratbegovic A Markovic N Ganibegovic Selimovic M. Molar incisor hypomineralisation in Bosnia and Herzegovina: aetiology and clinical consequences in medium caries activity population. Eur Arch Paediatr Dent. (2007) 8:189–94. 10.1007/BF03262595 18076849

[39] Whatling R Fearne JM. Molar incisor hypomineralization: a study of aetiological factors in a group of UK children. Int J Paediatr Dent. (2008) 18:155–62. 10.1111/j.1365-263X.2007.00901.x 18384347

[40] Wuollet E Laisi S Salmela E Ess A Alaluusua S. Molar-incisor hypomineralization and the association with childhood illnesses and antibiotics in a group of Finnish children. Acta Odontol Scand. (2016) 74:416–22. 10.3109/00016357.2016.1172342 27140829

[41] Wells GA Shea B O'Connell D Peterson J Welch V Losos M . The Newcastle-Ottawa Scale (NOS) for assessing the quality of nonrandomised studies in meta-analyses (2000).

[42] Higgins JP Thomas J Chandler J Cumpston M Li T Page MJ . Cochrane Handbook for Systematic Reviews of Interventions. John Wiley and Sons. (2019). 10.1002/9781119536604 31643080 PMC10284251

[43] GRADEpro. GDT: GRADEpro Guideline Development Tool. McMaster University (developed by Evidence Prime, Inc.). (2020). Available online at: https://www.gradepro.org/

[44] Loli D Costacurta M Maturo P Docimo R. Correlation between aerosol therapy in early childhood and molar incisor hypomineralisation. Eur J Paediatr Dent. (2015) 16:73–7. Available online at: https://art.torvergata.it/bitstream/2108/122375/2/MIH.pdf 25793958

[45] Lima LRS Pereira AS de Moura MS Lima CCB Paiva SM Moura LFAD . de Lima M. Pre-term birth and asthma is associated with hypomineralized second primary molars in pre-schoolers: a population-based study. Int J Paediatr Dent. (2020) 30:193–201. 10.1111/ipd.12584 31677213

[46] Mejía JD Restrepo M González S Álvarez LG Santos-Pinto L Escobar A. Molar incisor hypomineralization in colombia: prevalence, severity and associated risk factors. J Clin Pediatr Dent. (2019) 43:185–9. 10.17796/1053-4625-43.3.7 30964726

[47] Peker S Mete S Gokdemir Y Karadag B Kargul B. Related factors of dental caries and molar incisor hypomineralisation in a group of children with cystic fibrosis. Eur Arch Paediatr Dent. (2014) 15:275–80. 10.1007/s40368-014-0112-5 24569937

[48] Welbury RR. Paediatric Dentistry. Oxford University Press (1997). p. 11–2.

[49] Simmer JP. Dental enamel formation and its impact on clinical dentistry. J Dent Educ. (2001) 65:896–905. 10.1002/j.0022-0337.2001.65.9.tb03438.x 11569606

[50] Fearne J Anderson P Davis GR. 3D X-ray microscopic study of the extent of variations in enamel density in first permanent molars with idiopathic enamel hypomineralisation. Br Dent J. (2004) 196:634–8. 10.1038/sj.bdj.4811282 15153976

[51] Chawla N Messer LB Silva M. Clinical studies on molar-incisor-hypomineralisation part 2: development of a severity index. Eur Arch Paediatr Dent. (2008) 9:191–9. 10.1007/BF03262635 19054472

[52] Soviero V Haubek D Trindade C Poulsen S. Prevalence and distribution of demarcated opacities and their sequelae in permanent 1st molars and incisors in 7 to 13-year-old Brazilian children. Acta Odontol Scand. (2009) 67:170–5. 10.1080/00016350902758607 19253064

[53] Johnson D Kreji C Hack M Fanaroff A. Distribution of enamel defects and the association with respiratory distress in very low birth weight infants. J Dent Res. (1984) 63:59–64. 10.1177/00220345840630011401 6582082

[54] Koch G Hallonsten AL Ludvigsson N Hansson BO Holst A Ullbro C. Epidemiologic study of idiopathic enamel hypomineralization in permanent teeth of Swedish children. Comm Dent Oral Epidemiol. (1987) 15:279–85. 10.1111/j.1600-0528.1987.tb00538.x 3477361

[55] Whitford GM Angmar-Mansson B. Fluorosis-like effects of acidosis, but not NH4+ on rat incisor enamel. Caries Res. (1995) 29:20–5. 10.1159/000262035 7867046

[56] Sui W Boyd C Wright JT. Altered pH regulation during enamel development in the cystic fibrosis mouse incisor. J Dent Res. (2003) 82:388–92. 10.1177/154405910308200512 12709507

[57] Perez VA Mangum JE Hubbard MJ. Pathogenesis of molar hypomineralisation: aged albumin demarcates chalky regions of hypomineralised enamel. Front Physiol. (2020) 11:579015. 10.3389/fphys.2020.579015 33101060 PMC7556231

[58] Williams R Perez VA Mangum JE Hubbard MJ. Pathogenesis of molar hypomineralisation: hypomineralised 6-year molars contain traces of fetal serum albumin. Front Physiol. (2020) 11:619. 10.3389/fphys.2020.00619 32595522 PMC7303361

[59] Hubbard MJ Mangum JE Perez VA Williams R. A Breakthrough in understanding the pathogenesis of molar hypomineralisation: the mineralisation-poisoning model. Front Physiol. (2021) 12:802833. 10.3389/fphys.2021.802833 34992550 PMC8724775

[60] Haddad AE. (2001). A erupção dos dentes decí*duos e sua relação com o crescimento somático [Tese – Doutorado]*. São Paulo: Universidade de São Paulo (2001).

[61] Wong DL. Enfermagem Pediátrica - elementos essenciais à intervenção efetiva. Rio de Janeiro: Guanabara Koogan (2005).

[62] Dworkin PH. The National Medical Series for Independ Study. 3th ed. Rio de Janeiro: Guanabara Koogan (2001).

[63] Esperança AC Cavalcante RB Marcolino C. Estudo da demanda espontânea em uma unidade de saúde de saúde da família de uma cidade de médio porte do interior de Minas Gerais. Reme Rev Min Enferm. (2006) 10:30–6. Available online at: https://cdn.publisher.gn1.link/reme.org.br/pdf/v10n1a06.pdf

[64] Benício MHD'A Cardoso MRA Gouveia ND Monteiro CA. Tendência secular da doença respiratória na infância na cidade de São Paulo (1984-1996). Rev Saúde Pública. (2000) 34:91–101. 10.1590/S0034-89102000000700012 11434324

[65] Atallah AN Castro AA. Revisão sistemática da literatura e metanálise. Medicina baseada em evidências: fundamentos da pesquisa clí*nica São Paulo*. Lemos-Editorial (1998). p. 42–8.

[66] Mulrow CD. Rationale for systematic reviews. BMJ. (1994) 309:597–9. 10.1136/bmj.309.6954.597 8086953 PMC2541393

[67] Clarke M Horton R. Bringing it all together: lancet-cochrane collaborate on systematic reviews. Lancet. (2001) 357:1728. 10.1016/S0140-6736(00)04934-5 11403806

[68] Murad MH Asi N Alsawas M Alahdab F. New evidence pyramid. Evid Based Med. (2016) 21:125–7. 10.1136/ebmed-2016-110401 27339128 PMC4975798

